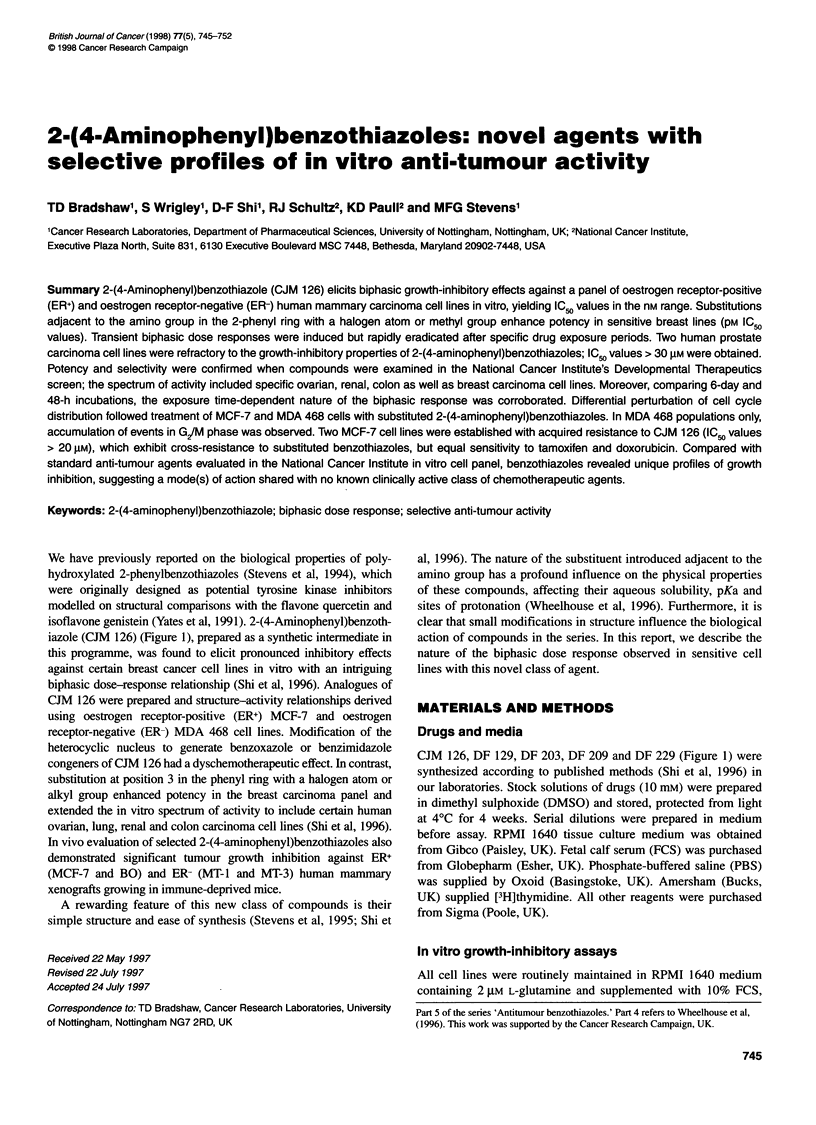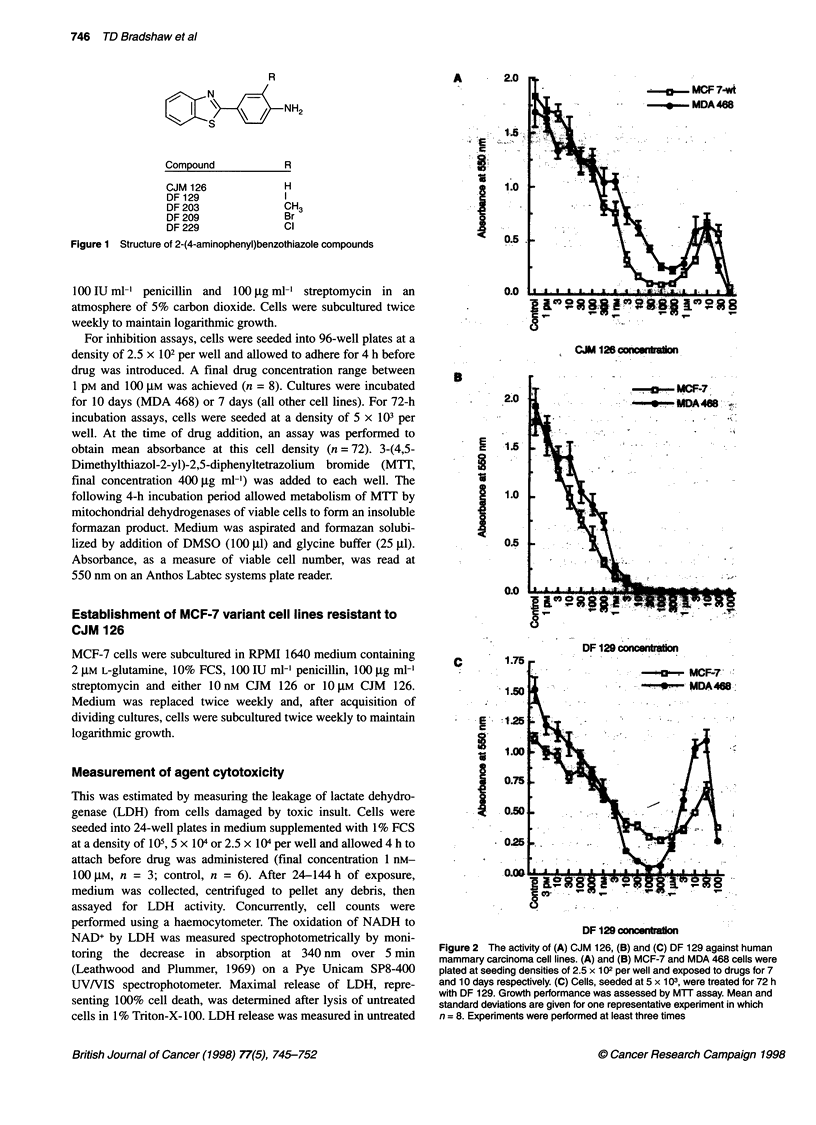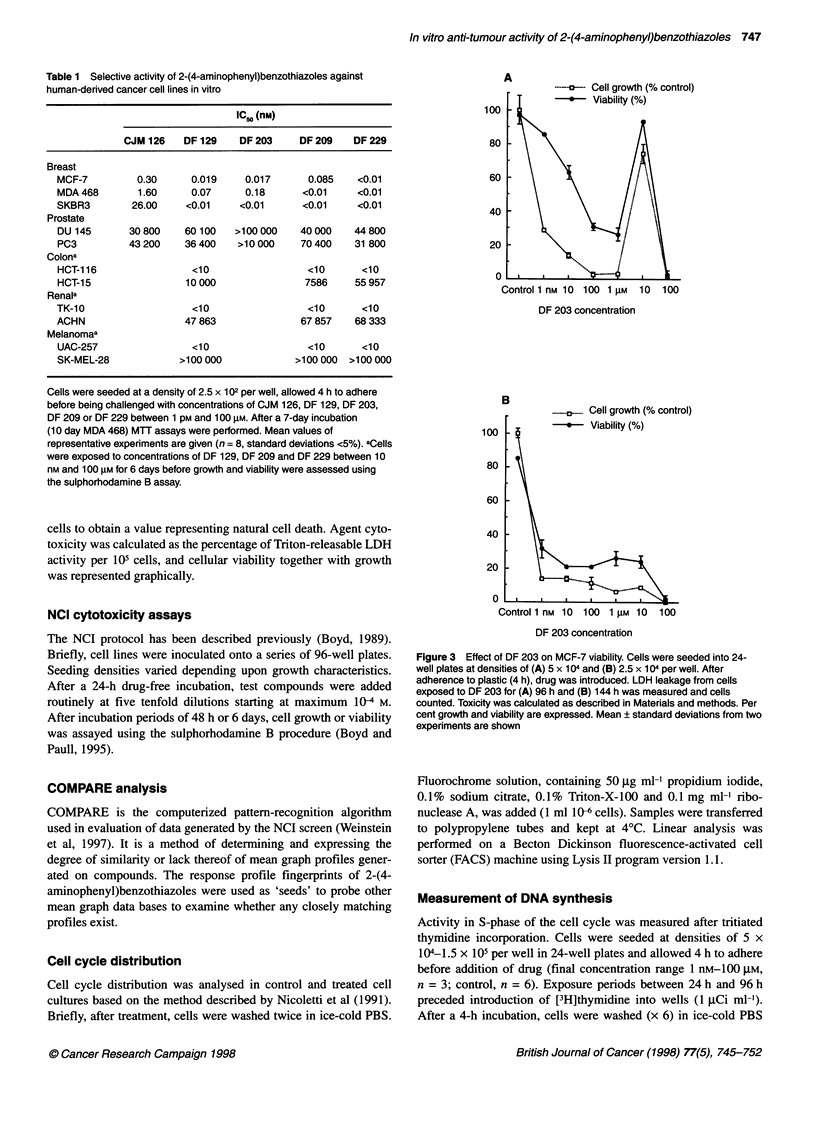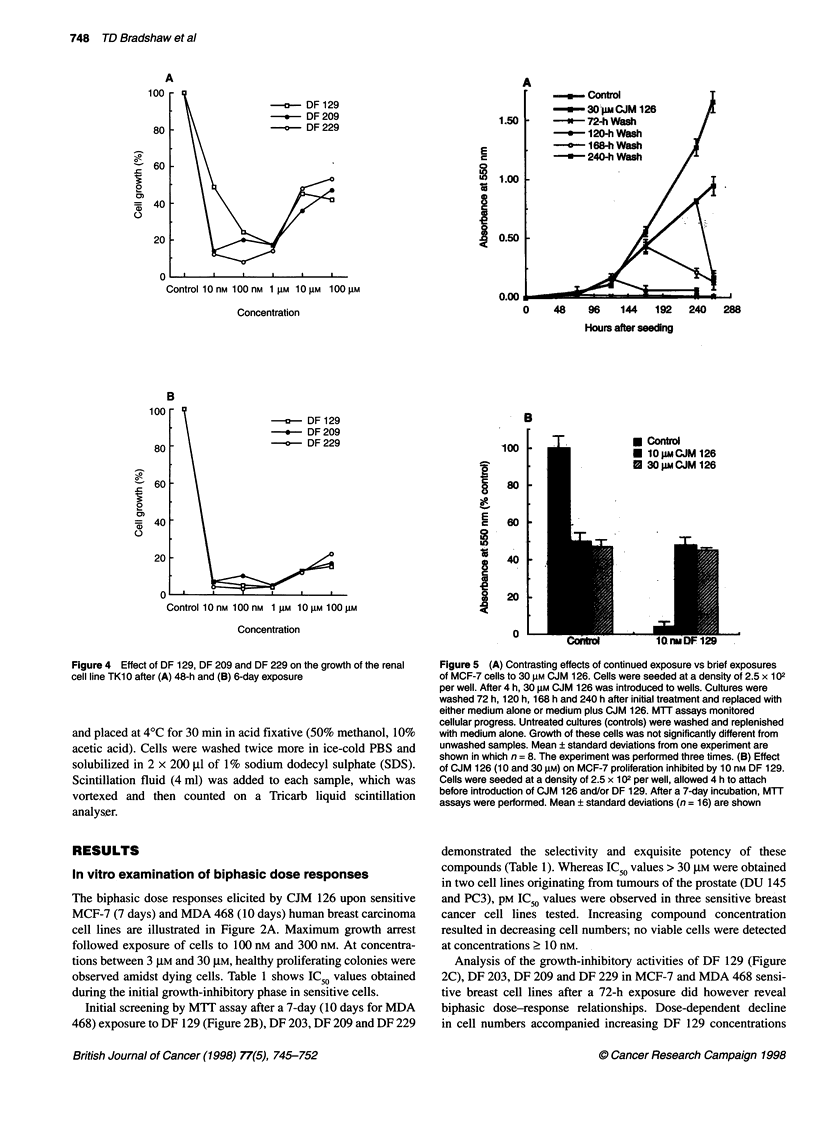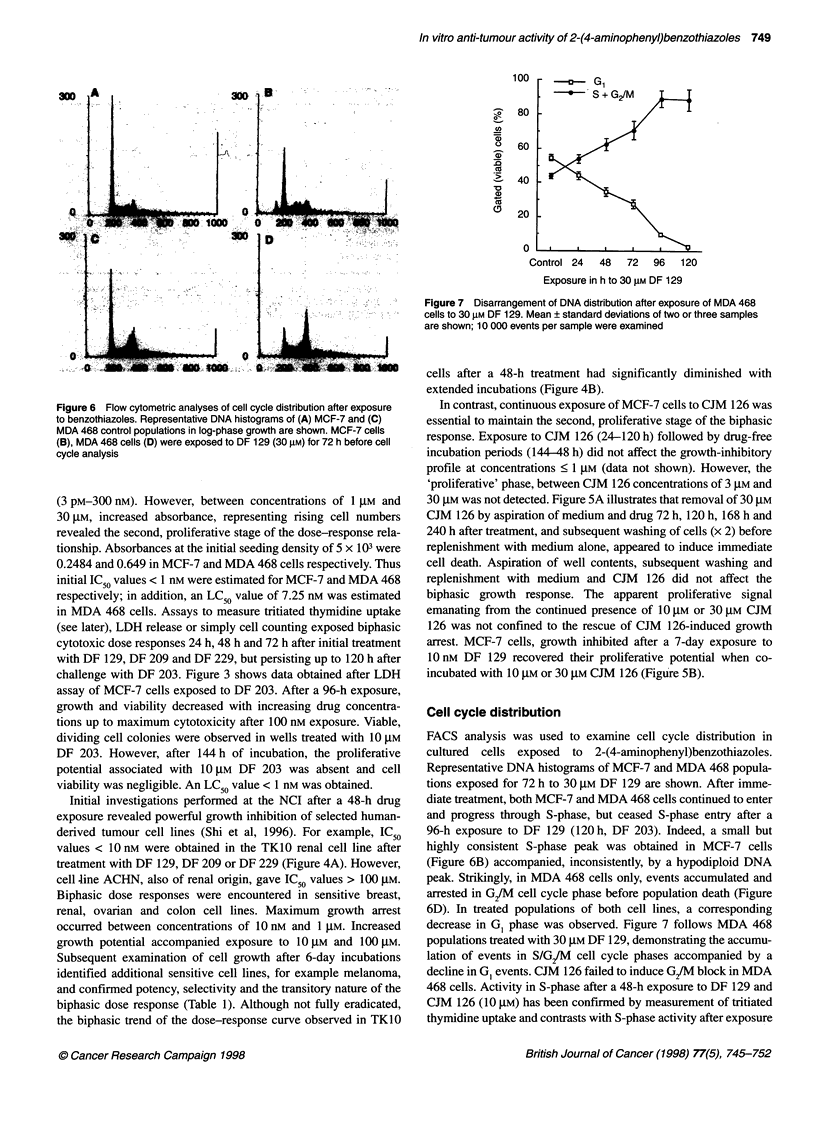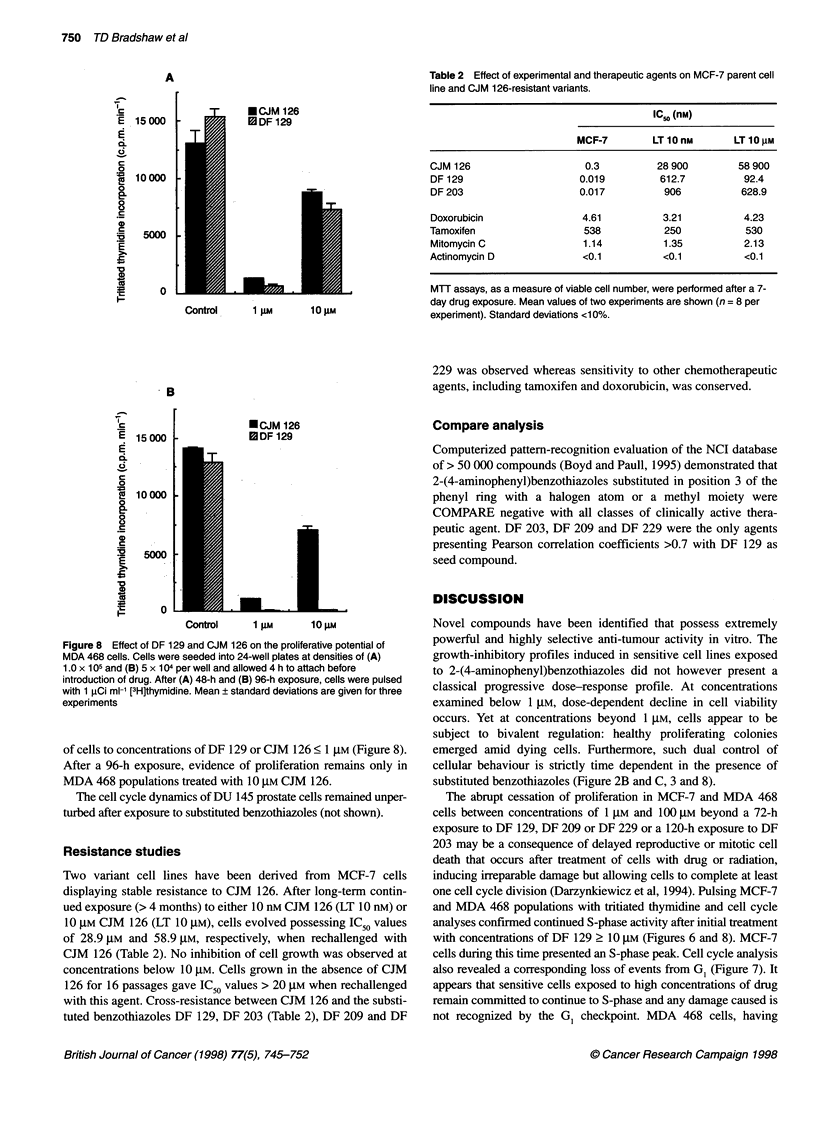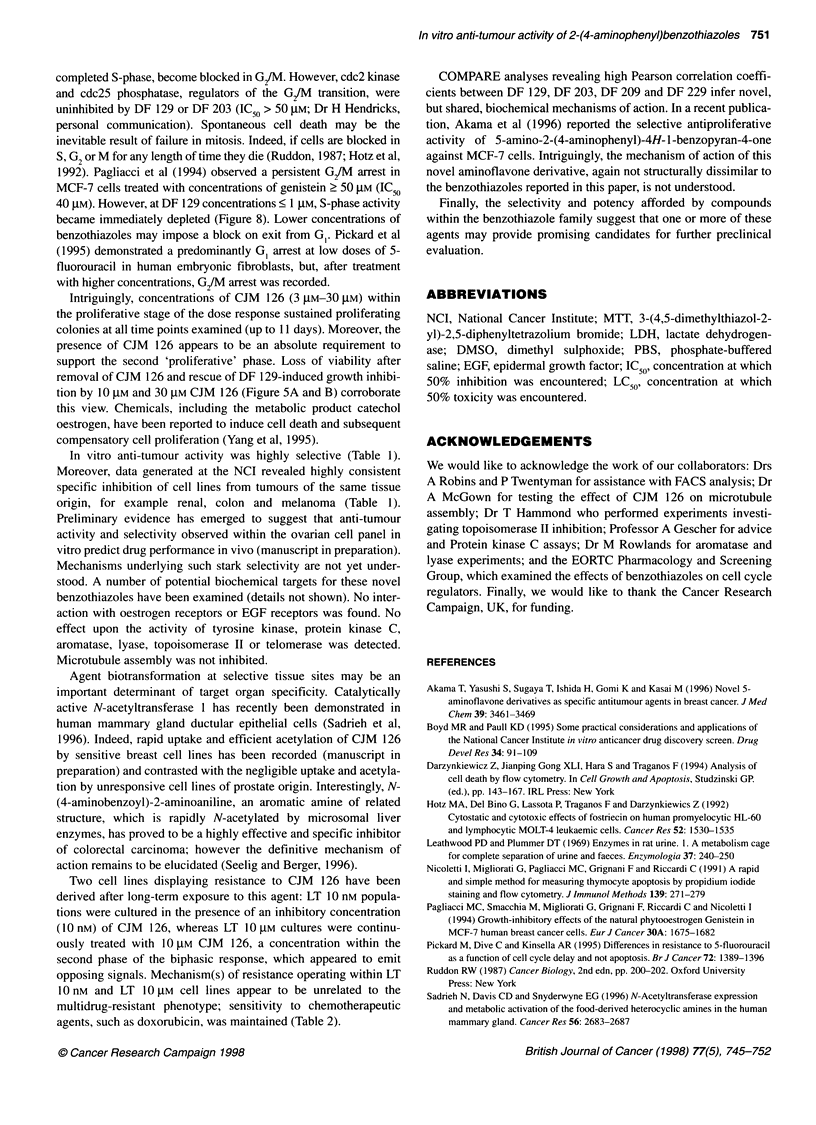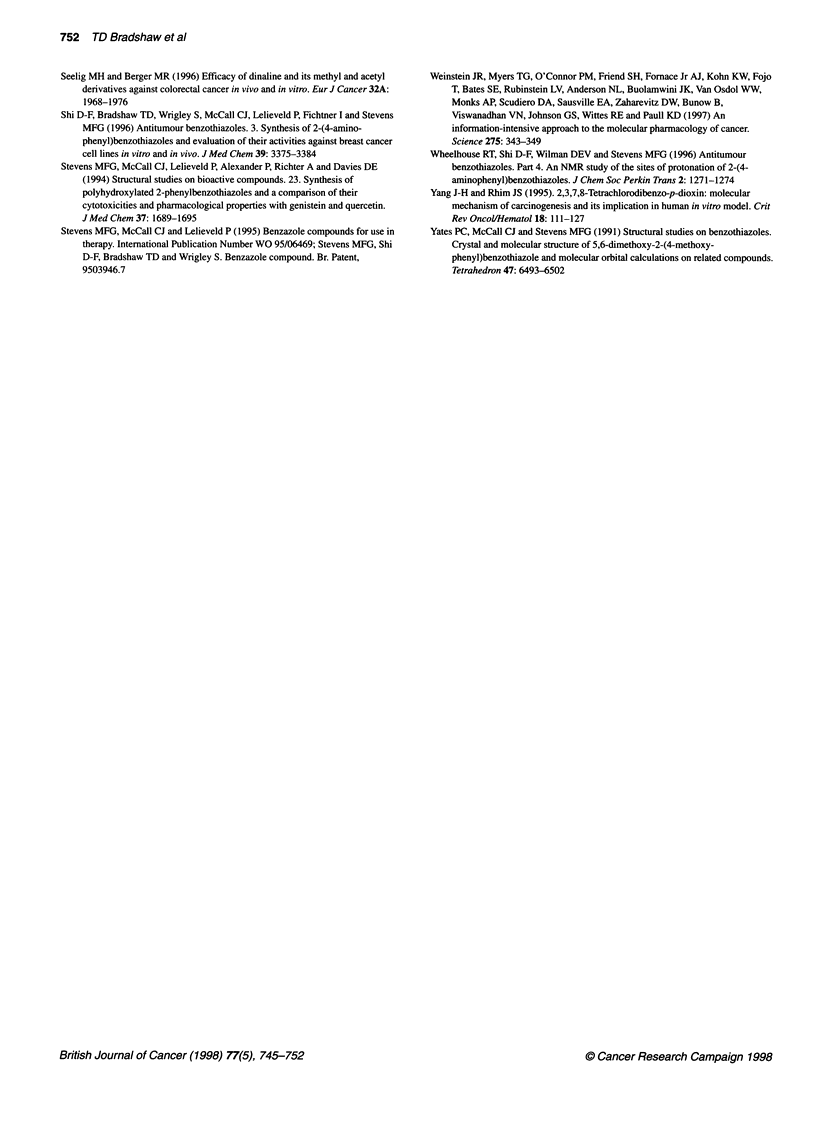# 2-(4-Aminophenyl)benzothiazoles: novel agents with selective profiles of in vitro anti-tumour activity.

**DOI:** 10.1038/bjc.1998.122

**Published:** 1998-03

**Authors:** T. D. Bradshaw, S. Wrigley, D. F. Shi, R. J. Schultz, K. D. Paull, M. F. Stevens

**Affiliations:** Department of Pharmaceutical Sciences, University of Nottingham, UK.

## Abstract

2-(4-Aminophenyl)benzothiazole (CJM 126) elicits biphasic growth-inhibitory effects against a panel of oestrogen receptor-positive (ER+) and oestrogen receptor-negative (ER-) human mammary carcinoma cell lines in vitro, yielding IC50 values in the nM range. Substitutions adjacent to the amino group in the 2-phenyl ring with a halogen atom or methyl group enhance potency in sensitive breast lines (pM IC50 values). Transient biphasic dose responses were induced but rapidly eradicated after specific drug exposure periods. Two human prostate carcinoma cell lines were refractory to the growth-inhibitory properties of 2-(4-aminophenyl)benzothiazoles; IC50 values > 30 microM were obtained. Potency and selectivity were confirmed when compounds were examined in the National Cancer Institute's Developmental Therapeutics screen; the spectrum of activity included specific ovarian, renal, colon as well as breast carcinoma cell lines. Moreover, comparing 6-day and 48-h incubations, the exposure time-dependent nature of the biphasic response was corroborated. Differential perturbation of cell cycle distribution followed treatment of MCF-7 and MDA 468 cells with substituted 2-(4-aminophenyl)benzothiazoles. In MDA 468 populations only, accumulation of events in G2/M phase was observed. Two MCF-7 cell lines were established with acquired resistance to CJM 126 (IC50 values > 20 microM), which exhibit cross-resistance to substituted benzothiazoles, but equal sensitivity to tamoxifen and doxorubicin. Compared with standard anti-tumour agents evaluated in the National Cancer Institute in vitro cell panel, benzothiazoles revealed unique profiles of growth inhibition, suggesting a mode(s) of action shared with no known clinically active class of chemotherapeutic agents.


British Journal of Cancer (1998) 77(5), 745–752
© 1998 Cancer Research Campaign

### 2-(4-Aminophenyl)benzothiazoles: novel agents with selective profiles of in vitro anti-tumour activity

**TD Bradshaw[1], S Wrigley[1], D-F Shi[1], RJ Schultz[2], KD Paull[2] and MFG Stevens[1]**

[1]Cancer Research Laboratories, Department of Pharmaceutical Sciences, University of Nottingham, Nottingham, UK; [2]National Cancer Institute, Executive Plaza North, Suite 831, 6130 Executive Boulevard MSC 7448, Bethesda, Maryland 20902-7448, USA

**Summary** 2-(4-Aminophenyl)benzothiazole (CJM 126) elicits biphasic growth-inhibitory effects against a panel of oestrogen receptor-positive ($ER^+$) and oestrogen receptor-negative ($ER^-$) human mammary carcinoma cell lines in vitro, yielding $IC_{50}$ values in the nM range. Substitutions adjacent to the amino group in the 2-phenyl ring with a halogen atom or methyl group enhance potency in sensitive breast lines (pM $IC_{50}$ values). Transient biphasic dose responses were induced but rapidly eradicated after specific drug exposure periods. Two human prostate carcinoma cell lines were refractory to the growth-inhibitory properties of 2-(4-aminophenyl)benzothiazoles; $IC_{50}$ values > 30 μM were obtained. Potency and selectivity were confirmed when compounds were examined in the National Cancer Institute's Developmental Therapeutics screen; the spectrum of activity included specific ovarian, renal, colon as well as breast carcinoma cell lines. Moreover, comparing 6-day and 48-h incubations, the exposure time-dependent nature of the biphasic response was corroborated. Differential perturbation of cell cycle distribution followed treatment of MCF-7 and MDA 468 cells with substituted 2-(4-aminophenyl)benzothiazoles. In MDA 468 populations only, accumulation of events in $G_2$/M phase was observed. Two MCF-7 cell lines were established with acquired resistance to CJM 126 ($IC_{50}$ values > 20 μM), which exhibit cross-resistance to substituted benzothiazoles, but equal sensitivity to tamoxifen and doxorubicin. Compared with standard anti-tumour agents evaluated in the National Cancer Institute in vitro cell panel, benzothiazoles revealed unique profiles of growth inhibition, suggesting a mode(s) of action shared with no known clinically active class of chemotherapeutic agents.

**Keywords:** 2-(4-aminophenyl)benzothiazole; biphasic dose response; selective anti-tumour activity

We have previously reported on the biological properties of polyhydroxylated 2-phenylbenzothiazoles (Stevens et al, 1994), which were originally designed as potential tyrosine kinase inhibitors modelled on structural comparisons with the flavone quercetin and isoflavone genistein (Yates et al, 1991). 2-(4-Aminophenyl)benzothiazole (CJM 126) (Figure 1), prepared as a synthetic intermediate in this programme, was found to elicit pronounced inhibitory effects against certain breast cancer cell lines in vitro with an intriguing biphasic dose–response relationship (Shi et al, 1996). Analogues of CJM 126 were prepared and structure–activity relationships derived using oestrogen receptor-positive ($ER^+$) MCF-7 and oestrogen receptor-negative ($ER^-$) MDA 468 cell lines. Modification of the heterocyclic nucleus to generate benzoxazole or benzimidazole congeners of CJM 126 had a dyschemotherapeutic effect. In contrast, substitution at position 3 in the phenyl ring with a halogen atom or alkyl group enhanced potency in the breast carcinoma panel and extended the in vitro spectrum of activity to include certain human ovarian, lung, renal and colon carcinoma cell lines (Shi et al, 1996). In vivo evaluation of selected 2-(4-aminophenyl)benzothiazoles also demonstrated significant tumour growth inhibition against $ER^+$ (MCF-7 and BO) and $ER^-$ (MT-1 and MT-3) human mammary xenografts growing in immune-deprived mice.

A rewarding feature of this new class of compounds is their simple structure and ease of synthesis (Stevens et al, 1995; Shi et al, 1996). The nature of the substituent introduced adjacent to the amino group has a profound influence on the physical properties of these compounds, affecting their aqueous solubility, p*K*a and sites of protonation (Wheelhouse et al, 1996). Furthermore, it is clear that small modifications in structure influence the biological action of compounds in the series. In this report, we describe the nature of the biphasic dose response observed in sensitive cell lines with this novel class of agent.

*Received 22 May 1997*
*Revised 22 July 1997*
*Accepted 24 July 1997*

*Correspondence to:* TD Bradshaw, Cancer Research Laboratories, University of Nottingham, Nottingham NG7 2RD, UK

### MATERIALS AND METHODS

### Drugs and media

CJM 126, DF 129, DF 203, DF 209 and DF 229 (Figure 1) were synthesized according to published methods (Shi et al, 1996) in our laboratories. Stock solutions of drugs (10 mM) were prepared in dimethyl sulphoxide (DMSO) and stored, protected from light at 4°C for 4 weeks. Serial dilutions were prepared in medium before assay. RPMI 1640 tissue culture medium was obtained from Gibco (Paisley, UK). Fetal calf serum (FCS) was purchased from Globepharm (Esher, UK). Phosphate-buffered saline (PBS) was supplied by Oxoid (Basingstoke, UK). Amersham (Bucks, UK) supplied [$^3$H]thymidine. All other reagents were purchased from Sigma (Poole, UK).

### In vitro growth-inhibitory assays

All cell lines were routinely maintained in RPMI 1640 medium containing 2 μM L-glutamine and supplemented with 10% FCS,

Part 5 of the series 'Antitumour benzothiazoles.' Part 4 refers to Wheelhouse et al, (1996). This work was supported by the Cancer Research Campaign, UK.

| Compound | R |
|---|---|
| CJM 126 | H |
| DF 129 | I |
| DF 203 | $CH_3$ |
| DF 209 | Br |
| DF 229 | Cl |

**Figure 1** Structure of 2-(4-aminophenyl)benzothiazole compounds

100 IU $ml^{-1}$ penicillin and 100 µg $ml^{-1}$ streptomycin in an atmosphere of 5% carbon dioxide. Cells were subcultured twice weekly to maintain logarithmic growth.

For inhibition assays, cells were seeded into 96-well plates at a density of $2.5 \times 10^2$ per well and allowed to adhere for 4 h before drug was introduced. A final drug concentration range between 1 pM and 100 µM was achieved ($n$ = 8). Cultures were incubated for 10 days (MDA 468) or 7 days (all other cell lines). For 72-h incubation assays, cells were seeded at a density of $5 \times 10^3$ per well. At the time of drug addition, an assay was performed to obtain mean absorbance at this cell density ($n$ = 72). 3-(4,5-Dimethylthiazol-2-yl)-2,5-diphenyltetrazolium bromide (MTT, final concentration 400 µg $ml^{-1}$) was added to each well. The following 4-h incubation period allowed metabolism of MTT by mitochondrial dehydrogenases of viable cells to form an insoluble formazan product. Medium was aspirated and formazan solubilized by addition of DMSO (100 µl) and glycine buffer (25 µl). Absorbance, as a measure of viable cell number, was read at 550 nm on an Anthos Labtec systems plate reader.

### Establishment of MCF-7 variant cell lines resistant to CJM 126

MCF-7 cells were subcultured in RPMI 1640 medium containing 2 µM L-glutamine, 10% FCS, 100 IU $ml^{-1}$ penicillin, 100 µg $ml^{-1}$ streptomycin and either 10 nM CJM 126 or 10 µM CJM 126. Medium was replaced twice weekly and, after acquisition of dividing cultures, cells were subcultured twice weekly to maintain logarithmic growth.

### Measurement of agent cytotoxicity

This was estimated by measuring the leakage of lactate dehydrogenase (LDH) from cells damaged by toxic insult. Cells were seeded into 24-well plates in medium supplemented with 1% FCS at a density of $10^5$, $5 \times 10^4$ or $2.5 \times 10^4$ per well and allowed 4 h to attach before drug was administered (final concentration 1 nM–100 µM, $n$ = 3; control, $n$ = 6). After 24–144 h of exposure, medium was collected, centrifuged to pellet any debris, then assayed for LDH activity. Concurrently, cell counts were performed using a haemocytometer. The oxidation of NADH to $NAD^+$ by LDH was measured spectrophotometrically by monitoring the decrease in absorption at 340 nm over 5 min (Leathwood and Plummer, 1969) on a Pye Unicam SP8-400 UV/VIS spectrophotometer. Maximal release of LDH, representing 100% cell death, was determined after lysis of untreated cells in 1% Triton-X-100. LDH release was measured in untreated

**Figure 2** The activity of (**A**) CJM 126, (**B**) and (**C**) DF 129 against human mammary carcinoma cell lines. (**A**) and (**B**) MCF-7 and MDA 468 cells were plated at seeding densities of $2.5 \times 10^2$ per well and exposed to drugs for 7 and 10 days respectively. (**C**) Cells, seeded at $5 \times 10^3$, were treated for 72 h with DF 129. Growth performance was assessed by MTT assay. Mean and standard deviations are given for one representative experiment in which $n$ = 8. Experiments were performed at least three times

© Cancer Research Campaign 1998

**Table 1** Selective activity of 2-(4-aminophenyl)benzothiazoles against human-derived cancer cell lines in vitro

| | IC$_{50}$ (nM) | | | | |
|---|---|---|---|---|---|
| | **CJM 126** | **DF 129** | **DF 203** | **DF 209** | **DF 229** |
| Breast | | | | | |
| MCF-7 | 0.30 | 0.019 | 0.017 | 0.085 | <0.01 |
| MDA 468 | 1.60 | 0.07 | 0.18 | <0.01 | <0.01 |
| SKBR3 | 26.00 | <0.01 | <0.01 | <0.01 | <0.01 |
| Prostate | | | | | |
| DU 145 | 30 800 | 60 100 | >100 000 | 40 000 | 44 800 |
| PC3 | 43 200 | 36 400 | >10 000 | 70 400 | 31 800 |
| Colon[a] | | | | | |
| HCT-116 | | <10 | | <10 | <10 |
| HCT-15 | | 10 000 | | 7586 | 55 957 |
| Renal[a] | | | | | |
| TK-10 | | <10 | | <10 | <10 |
| ACHN | | 47 863 | | 67 857 | 68 333 |
| Melanoma[a] | | | | | |
| UAC-257 | | <10 | | <10 | <10 |
| SK-MEL-28 | | >100 000 | | >100 000 | >100 000 |

Cells were seeded at a density of 2.5 × 10$^2$ per well, allowed 4 h to adhere before being challenged with concentrations of CJM 126, DF 129, DF 203, DF 209 or DF 229 between 1 pM and 100 μM. After a 7-day incubation (10 day MDA 468) MTT assays were performed. Mean values of representative experiments are given (*n* = 8, standard deviations <5%). [a]Cells were exposed to concentrations of DF 129, DF 209 and DF 229 between 10 nM and 100 μM for 6 days before growth and viability were assessed using the sulphorhodamine B assay.

cells to obtain a value representing natural cell death. Agent cytotoxicity was calculated as the percentage of Triton-releasable LDH activity per 10$^5$ cells, and cellular viability together with growth was represented graphically.

### NCI cytotoxicity assays

The NCI protocol has been described previously (Boyd, 1989). Briefly, cell lines were inoculated onto a series of 96-well plates. Seeding densities varied depending upon growth characteristics. After a 24-h drug-free incubation, test compounds were added routinely at five tenfold dilutions starting at maximum 10$^{-4}$ M. After incubation periods of 48 h or 6 days, cell growth or viability was assayed using the sulphorhodamine B procedure (Boyd and Paull, 1995).

### COMPARE analysis

COMPARE is the computerized pattern-recognition algorithm used in evaluation of data generated by the NCI screen (Weinstein et al, 1997). It is a method of determining and expressing the degree of similarity or lack thereof of mean graph profiles generated on compounds. The response profile fingerprints of 2-(4-aminophenyl)benzothiazoles were used as 'seeds' to probe other mean graph data bases to examine whether any closely matching profiles exist.

### Cell cycle distribution

Cell cycle distribution was analysed in control and treated cell cultures based on the method described by Nicoletti et al (1991). Briefly, after treatment, cells were washed twice in ice-cold PBS.

**Figure 3** Effect of DF 203 on MCF-7 viability. Cells were seeded into 24-well plates at densities of (**A**) 5 × 10$^4$ and (**B**) 2.5 × 10$^4$ per well. After adherence to plastic (4 h), drug was introduced. LDH leakage from cells exposed to DF 203 for (**A**) 96 h and (**B**) 144 h was measured and cells counted. Toxicity was calculated as described in Materials and methods. Per cent growth and viability are expressed. Mean ± standard deviations from two experiments are shown

Fluorochrome solution, containing 50 μg ml$^{-1}$ propidium iodide, 0.1% sodium citrate, 0.1% Triton-X-100 and 0.1 mg ml$^{-1}$ ribonuclease A, was added (1 ml 10$^{-6}$ cells). Samples were transferred to polypropylene tubes and kept at 4°C. Linear analysis was performed on a Becton Dickinson fluorescence-activated cell sorter (FACS) machine using Lysis II program version 1.1.

### Measurement of DNA synthesis

Activity in S-phase of the cell cycle was measured after tritiated thymidine incorporation. Cells were seeded at densities of 5 × 10$^4$–1.5 × 10$^5$ per well in 24-well plates and allowed 4 h to adhere before addition of drug (final concentration range 1 nM–100 μM, *n* = 3; control, *n* = 6). Exposure periods between 24 h and 96 h preceded introduction of [$^3$H]thymidine into wells (1 μCi ml$^{-1}$). After a 4-h incubation, cells were washed (× 6) in ice-cold PBS

**Figure 4** Effect of DF 129, DF 209 and DF 229 on the growth of the renal cell line TK10 after (**A**) 48-h and (**B**) 6-day exposure

**Figure 5** (**A**) Contrasting effects of continued exposure vs brief exposures of MCF-7 cells to 30 µM CJM 126. Cells were seeded at a density of 2.5 × $10^2$ per well. After 4 h, 30 µM CJM 126 was introduced to wells. Cultures were washed 72 h, 120 h, 168 h and 240 h after initial treatment and replaced with either medium alone or medium plus CJM 126. MTT assays monitored cellular progress. Untreated cultures (controls) were washed and replenished with medium alone. Growth of these cells was not significantly different from unwashed samples. Mean ± standard deviations from one experiment are shown in which $n$ = 8. The experiment was performed three times. (**B**) Effect of CJM 126 (10 and 30 µM) on MCF-7 proliferation inhibited by 10 nM DF 129. Cells were seeded at a density of 2.5 × $10^2$ per well, allowed 4 h to attach before introduction of CJM 126 and/or DF 129. After a 7-day incubation, MTT assays were performed. Mean ± standard deviations ($n$ = 16) are shown and placed at 4°C for 30 min in acid fixative (50% methanol, 10% acetic acid). Cells were washed twice more in ice-cold PBS and solubilized in 2 × 200 µl of 1% sodium dodecyl sulphate (SDS). Scintillation fluid (4 ml) was added to each sample, which was vortexed and then counted on a Tricarb liquid scintillation analyser.

### RESULTS

### In vitro examination of biphasic dose responses

The biphasic dose responses elicited by CJM 126 upon sensitive MCF-7 (7 days) and MDA 468 (10 days) human breast carcinoma cell lines are illustrated in Figure 2A. Maximum growth arrest followed exposure of cells to 100 nM and 300 nM. At concentrations between 3 µM and 30 µM, healthy proliferating colonies were observed amidst dying cells. Table 1 shows $IC_{50}$ values obtained during the initial growth-inhibitory phase in sensitive cells.

Initial screening by MTT assay after a 7-day (10 days for MDA 468) exposure to DF 129 (Figure 2B), DF 203, DF 209 and DF 229 demonstrated the selectivity and exquisite potency of these compounds (Table 1). Whereas $IC_{50}$ values > 30 µM were obtained in two cell lines originating from tumours of the prostate (DU 145 and PC3), pM $IC_{50}$ values were observed in three sensitive breast cancer cell lines tested. Increasing compound concentration resulted in decreasing cell numbers; no viable cells were detected at concentrations ≥ 10 nM.

Analysis of the growth-inhibitory activities of DF 129 (Figure 2C), DF 203, DF 209 and DF 229 in MCF-7 and MDA 468 sensitive breast cell lines after a 72-h exposure did however reveal biphasic dose–response relationships. Dose-dependent decline in cell numbers accompanied increasing DF 129 concentrations

© Cancer Research Campaign 1998

**Figure 6** Flow cytometric analyses of cell cycle distribution after exposure to benzothiazoles. Representative DNA histograms of (**A**) MCF-7 and (**C**) MDA 468 control populations in log-phase growth are shown. MCF-7 cells (**B**), MDA 468 cells (**D**) were exposed to DF 129 (30 µM) for 72 h before cell cycle analysis

**Figure 7** Disarrangement of DNA distribution after exposure of MDA 468 cells to 30 µM DF 129. Mean ± standard deviations of two or three samples are shown; 10 000 events per sample were examined

(3 pM–300 nM). However, between concentrations of 1 µM and 30 µM, increased absorbance, representing rising cell numbers revealed the second, proliferative stage of the dose–response relationship. Absorbances at the initial seeding density of $5 \times 10^3$ were 0.2484 and 0.649 in MCF-7 and MDA 468 cells respectively. Thus initial $IC_{50}$ values < 1 nM were estimated for MCF-7 and MDA 468 respectively; in addition, an $LC_{50}$ value of 7.25 nM was estimated in MDA 468 cells. Assays to measure tritiated thymidine uptake (see later), LDH release or simply cell counting exposed biphasic cytotoxic dose responses 24 h, 48 h and 72 h after initial treatment with DF 129, DF 209 and DF 229, but persisting up to 120 h after challenge with DF 203. Figure 3 shows data obtained after LDH assay of MCF-7 cells exposed to DF 203. After a 96-h exposure, growth and viability decreased with increasing drug concentrations up to maximum cytotoxicity after 100 nM exposure. Viable, dividing cell colonies were observed in wells treated with 10 µM DF 203. However, after 144 h of incubation, the proliferative potential associated with 10 µM DF 203 was absent and cell viability was negligible. An $LC_{50}$ value < 1 nM was obtained.

Initial investigations performed at the NCI after a 48-h drug exposure revealed powerful growth inhibition of selected human-derived tumour cell lines (Shi et al, 1996). For example, $IC_{50}$ values < 10 nM were obtained in the TK10 renal cell line after treatment with DF 129, DF 209 or DF 229 (Figure 4A). However, cell line ACHN, also of renal origin, gave $IC_{50}$ values > 100 µM. Biphasic dose responses were encountered in sensitive breast, renal, ovarian and colon cell lines. Maximum growth arrest occurred between concentrations of 10 nM and 1 µM. Increased growth potential accompanied exposure to 10 µM and 100 µM. Subsequent examination of cell growth after 6-day incubations identified additional sensitive cell lines, for example melanoma, and confirmed potency, selectivity and the transitory nature of the biphasic dose response (Table 1). Although not fully eradicated, the biphasic trend of the dose–response curve observed in TK10 cells after a 48-h treatment had significantly diminished with extended incubations (Figure 4B).

In contrast, continuous exposure of MCF-7 cells to CJM 126 was essential to maintain the second, proliferative stage of the biphasic response. Exposure to CJM 126 (24–120 h) followed by drug-free incubation periods (144–48 h) did not affect the growth-inhibitory profile at concentrations ≤ 1 µM (data not shown). However, the 'proliferative' phase, between CJM 126 concentrations of 3 µM and 30 µM was not detected. Figure 5A illustrates that removal of 30 µM CJM 126 by aspiration of medium and drug 72 h, 120 h, 168 h and 240 h after treatment, and subsequent washing of cells (× 2) before replenishment with medium alone, appeared to induce immediate cell death. Aspiration of well contents, subsequent washing and replenishment with medium and CJM 126 did not affect the biphasic growth response. The apparent proliferative signal emanating from the continued presence of 10 µM or 30 µM CJM 126 was not confined to the rescue of CJM 126-induced growth arrest. MCF-7 cells, growth inhibited after a 7-day exposure to 10 nM DF 129 recovered their proliferative potential when co-incubated with 10 µM or 30 µM CJM 126 (Figure 5B).

### Cell cycle distribution

FACS analysis was used to examine cell cycle distribution in cultured cells exposed to 2-(4-aminophenyl)benzothiazoles. Representative DNA histograms of MCF-7 and MDA 468 populations exposed for 72 h to 30 µM DF 129 are shown. After immediate treatment, both MCF-7 and MDA 468 cells continued to enter and progress through S-phase, but ceased S-phase entry after a 96-h exposure to DF 129 (120 h, DF 203). Indeed, a small but highly consistent S-phase peak was obtained in MCF-7 cells (Figure 6B) accompanied, inconsistently, by a hypodiploid DNA peak. Strikingly, in MDA 468 cells only, events accumulated and arrested in $G_2/M$ cell cycle phase before population death (Figure 6D). In treated populations of both cell lines, a corresponding decrease in $G_1$ phase was observed. Figure 7 follows MDA 468 populations treated with 30 µM DF 129, demonstrating the accumulation of events in $S/G_2/M$ cell cycle phases accompanied by a decline in $G_1$ events. CJM 126 failed to induce $G_2/M$ block in MDA 468 cells. Activity in S-phase after a 48-h exposure to DF 129 and CJM 126 (10 µM) has been confirmed by measurement of tritiated thymidine uptake and contrasts with S-phase activity after exposure

© Cancer Research Campaign 1998

**Figure 8** Effect of DF 129 and CJM 126 on the proliferative potential of MDA 468 cells. Cells were seeded into 24-well plates at densities of (**A**) $1.0 \times 10^5$ and (**B**) $5 \times 10^4$ per well and allowed 4 h to attach before introduction of drug. After (**A**) 48-h and (**B**) 96-h exposure, cells were pulsed with 1 μCi $ml^{-1}$ [$^3$H]thymidine. Mean ± standard deviations are given for three experiments of cells to concentrations of DF 129 or CJM 126 ≤ 1 μM (Figure 8). After a 96-h exposure, evidence of proliferation remains only in MDA 468 populations treated with 10 μM CJM 126.

The cell cycle dynamics of DU 145 prostate cells remained unperturbed after exposure to substituted benzothiazoles (not shown).

### Resistance studies

Two variant cell lines have been derived from MCF-7 cells displaying stable resistance to CJM 126. After long-term continued exposure (> 4 months) to either 10 nM CJM 126 (LT 10 nM) or 10 μM CJM 126 (LT 10 μM), cells evolved possessing $IC_{50}$ values of 28.9 μM and 58.9 μM, respectively, when rechallenged with CJM 126 (Table 2). No inhibition of cell growth was observed at concentrations below 10 μM. Cells grown in the absence of CJM 126 for 16 passages gave $IC_{50}$ values > 20 μM when rechallenged with this agent. Cross-resistance between CJM 126 and the substituted benzothiazoles DF 129, DF 203 (Table 2), DF 209 and DF 229 was observed whereas sensitivity to other chemotherapeutic agents, including tamoxifen and doxorubicin, was conserved.

**Table 2** Effect of experimental and therapeutic agents on MCF-7 parent cell line and CJM 126-resistant variants.

| | $IC_{50}$ (nM) | | |
|---|---|---|---|
| | MCF-7 | LT 10 nM | LT 10 μM |
| CJM 126 | 0.3 | 28 900 | 58 900 |
| DF 129 | 0.019 | 612.7 | 92.4 |
| DF 203 | 0.017 | 906 | 628.9 |
| Doxorubicin | 4.61 | 3.21 | 4.23 |
| Tamoxifen | 538 | 250 | 530 |
| Mitomycin C | 1.14 | 1.35 | 2.13 |
| Actinomycin D | <0.1 | <0.1 | <0.1 |

MTT assays, as a measure of viable cell number, were performed after a 7-day drug exposure. Mean values of two experiments are shown (*n* = 8 per experiment). Standard deviations <10%.

### Compare analysis

Computerized pattern-recognition evaluation of the NCI database of > 50 000 compounds (Boyd and Paull, 1995) demonstrated that 2-(4-aminophenyl)benzothiazoles substituted in position 3 of the phenyl ring with a halogen atom or a methyl moiety were COMPARE negative with all classes of clinically active therapeutic agent. DF 203, DF 209 and DF 229 were the only agents presenting Pearson correlation coefficients >0.7 with DF 129 as seed compound.

### DISCUSSION

Novel compounds have been identified that possess extremely powerful and highly selective anti-tumour activity in vitro. The growth-inhibitory profiles induced in sensitive cell lines exposed to 2-(4-aminophenyl)benzothiazoles did not however present a classical progressive dose–response profile. At concentrations examined below 1 μM, dose-dependent decline in cell viability occurs. Yet at concentrations beyond 1 μM, cells appear to be subject to bivalent regulation: healthy proliferating colonies emerged amid dying cells. Furthermore, such dual control of cellular behaviour is strictly time dependent in the presence of substituted benzothiazoles (Figure 2B and C, 3 and 8).

The abrupt cessation of proliferation in MCF-7 and MDA 468 cells between concentrations of 1 μM and 100 μM beyond a 72-h exposure to DF 129, DF 209 or DF 229 or a 120-h exposure to DF 203 may be a consequence of delayed reproductive or mitotic cell death that occurs after treatment of cells with drug or radiation, inducing irreparable damage but allowing cells to complete at least one cell cycle division (Darzynkiewicz et al, 1994). Pulsing MCF-7 and MDA 468 populations with tritiated thymidine and cell cycle analyses confirmed continued S-phase activity after initial treatment with concentrations of DF 129 ≥ 10 μM (Figures 6 and 8). MCF-7 cells during this time presented an S-phase peak. Cell cycle analysis also revealed a corresponding loss of events from $G_1$ (Figure 7). It appears that sensitive cells exposed to high concentrations of drug remain committed to continue to S-phase and any damage caused is not recognized by the $G_1$ checkpoint. MDA 468 cells, having

 © Cancer Research Campaign 1998

completed S-phase, become blocked in $G_2$/M. However, cdc2 kinase and cdc25 phosphatase, regulators of the $G_2$/M transition, were uninhibited by DF 129 or DF 203 ($IC_{50}$ > 50 μM; Dr H Hendricks, personal communication). Spontaneous cell death may be the inevitable result of failure in mitosis. Indeed, if cells are blocked in S, $G_2$ or M for any length of time they die (Ruddon, 1987; Hotz et al, 1992). Pagliacci et al (1994) observed a persistent $G_2$/M arrest in MCF-7 cells treated with concentrations of genistein ≥ 50 μM ($IC_{50}$ 40 μM). However, at DF 129 concentrations ≤ 1 μM, S-phase activity became immediately depleted (Figure 8). Lower concentrations of benzothiazoles may impose a block on exit from $G_1$. Pickard et al (1995) demonstrated a predominantly $G_1$ arrest at low doses of 5-fluorouracil in human embryonic fibroblasts, but, after treatment with higher concentrations, $G_2$/M arrest was recorded.

Intriguingly, concentrations of CJM 126 (3 μM–30 μM) within the proliferative stage of the dose response sustained proliferating colonies at all time points examined (up to 11 days). Moreover, the presence of CJM 126 appears to be an absolute requirement to support the second 'proliferative' phase. Loss of viability after removal of CJM 126 and rescue of DF 129-induced growth inhibition by 10 μM and 30 μM CJM 126 (Figure 5A and B) corroborate this view. Chemicals, including the metabolic product catechol oestrogen, have been reported to induce cell death and subsequent compensatory cell proliferation (Yang et al, 1995).

In vitro anti-tumour activity was highly selective (Table 1). Moreover, data generated at the NCI revealed highly consistent specific inhibition of cell lines from tumours of the same tissue origin, for example renal, colon and melanoma (Table 1). Preliminary evidence has emerged to suggest that anti-tumour activity and selectivity observed within the ovarian cell panel in vitro predict drug performance in vivo (manuscript in preparation). Mechanisms underlying such stark selectivity are not yet understood. A number of potential biochemical targets for these novel benzothiazoles have been examined (details not shown). No interaction with oestrogen receptors or EGF receptors was found. No effect upon the activity of tyrosine kinase, protein kinase C, aromatase, lyase, topoisomerase II or telomerase was detected. Microtubule assembly was not inhibited.

Agent biotransformation at selective tissue sites may be an important determinant of target organ specificity. Catalytically active *N*-acetyltransferase 1 has recently been demonstrated in human mammary gland ductular epithelial cells (Sadrieh et al, 1996). Indeed, rapid uptake and efficient acetylation of CJM 126 by sensitive breast cell lines has been recorded (manuscript in preparation) and contrasted with the negligible uptake and acetylation by unresponsive cell lines of prostate origin. Interestingly, *N*-(4-aminobenzoyl)-2-aminoaniline, an aromatic amine of related structure, which is rapidly *N*-acetylated by microsomal liver enzymes, has proved to be a highly effective and specific inhibitor of colorectal carcinoma; however the definitive mechanism of action remains to be elucidated (Seelig and Berger, 1996).

Two cell lines displaying resistance to CJM 126 have been derived after long-term exposure to this agent: LT 10 nM populations were cultured in the presence of an inhibitory concentration (10 nM) of CJM 126, whereas LT 10 μM cultures were continuously treated with 10 μM CJM 126, a concentration within the second phase of the biphasic response, which appeared to emit opposing signals. Mechanism(s) of resistance operating within LT 10 nM and LT 10 μM cell lines appear to be unrelated to the multidrug-resistant phenotype; sensitivity to chemotherapeutic agents, such as doxorubicin, was maintained (Table 2).

COMPARE analyses revealing high Pearson correlation coefficients between DF 129, DF 203, DF 209 and DF 229 infer novel, but shared, biochemical mechanisms of action. In a recent publication, Akama et al (1996) reported the selective antiproliferative activity of 5-amino-2-(4-aminophenyl)-4*H*-1-benzopyran-4-one against MCF-7 cells. Intriguingly, the mechanism of action of this novel aminoflavone derivative, again not structurally dissimilar to the benzothiazoles reported in this paper, is not understood.

Finally, the selectivity and potency afforded by compounds within the benzothiazole family suggest that one or more of these agents may provide promising candidates for further preclinical evaluation.

### ABBREVIATIONS

NCI, National Cancer Institute; MTT, 3-(4,5-dimethylthiazol-2-yl)-2,5-diphenyltetrazolium bromide; LDH, lactate dehydrogenase; DMSO, dimethyl sulphoxide; PBS, phosphate-buffered saline; EGF, epidermal growth factor; $IC_{50}$, concentration at which 50% inhibition was encountered; $LC_{50}$, concentration at which 50% toxicity was encountered.

### ACKNOWLEDGEMENTS

We would like to acknowledge the work of our collaborators: Drs A Robins and P Twentyman for assistance with FACS analysis; Dr A McGown for testing the effect of CJM 126 on microtubule assembly; Dr T Hammond who performed experiments investigating topoisomerase II inhibition; Professor A Gescher for advice and Protein kinase C assays; Dr M Rowlands for aromatase and lyase experiments; and the EORTC Pharmacology and Screening Group, which examined the effects of benzothiazoles on cell cycle regulators. Finally, we would like to thank the Cancer Research Campaign, UK, for funding.

### REFERENCES

Akama T, Yasushi S, Sugaya T, Ishida H, Gomi K and Kasai M (1996) Novel 5-aminoflavone derivatives as specific antitumour agents in breast cancer. *J Med Chem* **39**: 3461–3469

Boyd MR and Paull KD (1995) Some practical considerations and applications of the National Cancer Institute *in vitro* anticancer drug discovery screen. *Drug Devel Res* **34**: 91–109

Darzynkiewicz Z, Jianping Gong XLI, Hara S and Traganos F (1994) Analysis of cell death by flow cytometry. In *Cell Growth and Apoptosis*, Studzinski GP. (ed.), pp. 143–167. IRL Press: New York

Hotz MA, Del Bino G, Lassota P, Traganos F and Darzynkiewics Z (1992) Cytostatic and cytotoxic effects of fostriecin on human promyelocytic HL-60 and lymphocytic MOLT-4 leukaemic cells. *Cancer Res* **52**: 1530–1535

Leathwood PD and Plummer DT (1969) Enzymes in rat urine. 1. A metabolism cage for complete separation of urine and faeces. *Enzymologia* **37**: 240–250

Nicoletti I, Migliorati G, Pagliacci MC, Grignani F and Riccardi C (1991) A rapid and simple method for measuring thymocyte apoptosis by propidium iodide staining and flow cytometry. *J Immunol Methods* **139**: 271–279

Pagliacci MC, Smacchia M, Migliorati G, Grignani F, Riccardi C and Nicoletti I (1994) Growth-inhibitory effects of the natural phytooestrogen Genistein in MCF-7 human breast cancer cells. *Eur J Cancer* **30A**: 1675–1682

Pickard M, Dive C and Kinsella AR (1995) Differences in resistance to 5-fluorouracil as a function of cell cycle delay and not apoptosis. *Br J Cancer* **72**: 1389–1396

Ruddon RW (1987) *Cancer Biology*, 2nd edn, pp. 200–202. Oxford University Press: New York

Sadrieh N, Davis CD and Snyderwyne EG (1996) *N*-Acetyltransferase expression and metabolic activation of the food-derived heterocyclic amines in the human mammary gland. *Cancer Res* **56**: 2683–2687

© Cancer Research Campaign 1998

Seelig MH and Berger MR (1996) Efficacy of dinaline and its methyl and acetyl derivatives against colorectal cancer *in vivo* and *in vitro*. *Eur J Cancer* **32A**: 1968–1976

Shi D-F, Bradshaw TD, Wrigley S, McCall CJ, Lelieveld P, Fichtner I and Stevens MFG (1996) Antitumour benzothiazoles. 3. Synthesis of 2-(4-aminophenyl)benzothiazoles and evaluation of their activities against breast cancer cell lines *in vitro* and *in vivo*. *J Med Chem* **39**: 3375–3384

Stevens MFG, McCall CJ, Lelieveld P, Alexander P, Richter A and Davies DE (1994) Structural studies on bioactive compounds. 23. Synthesis of polyhydroxylated 2-phenylbenzothiazoles and a comparison of their cytotoxicities and pharmacological properties with genistein and quercetin. *J Med Chem* **37**: 1689–1695

Stevens MFG, McCall CJ and Lelieveld P (1995) Benzazole compounds for use in therapy. International Publication Number WO 95/06469; Stevens MFG, Shi D-F, Bradshaw TD and Wrigley S. Benzazole compound. Br. Patent, 9503946.7

Weinstein JR, Myers TG, O'Connor PM, Friend SH, Fornace Jr AJ, Kohn KW, Fojo T, Bates SE, Rubinstein LV, Anderson NL, Buolamwini JK, Van Osdol WW, Monks AP, Scudiero DA, Sausville EA, Zaharevitz DW, Bunow B, Viswanadhan VN, Johnson GS, Wittes RE and Paull KD (1997) An information-intensive approach to the molecular pharmacology of cancer. *Science* **275**: 343–349

Wheelhouse RT, Shi D-F, Wilman DEV and Stevens MFG (1996) Antitumour benzothiazoles. Part 4. An NMR study of the sites of protonation of 2-(4-aminophenyl)benzothiazoles. *J Chem Soc Perkin Trans* **2**: 1271–1274

Yang J-H and Rhim JS (1995). 2,3,7,8-Tetrachlorodibenzo-*p*-dioxin: molecular mechanism of carcinogenesis and its implication in human *in vitro* model. *Crit Rev Oncol/Hematol* **18**: 111–127

Yates PC, McCall CJ and Stevens MFG (1991) Structural studies on benzothiazoles. Crystal and molecular structure of 5,6-dimethoxy-2-(4-methoxyphenyl)benzothiazole and molecular orbital calculations on related compounds. *Tetrahedron* **47**: 6493–6502

© Cancer Research Campaign 1998